# Nurse-Driven Transitions Program Provides High Value End-of-Life Care to Veterans With Serious Illness

**DOI:** 10.1093/geroni/igaa057.789

**Published:** 2020-12-16

**Authors:** Jane Driver, Barbara Hayes, Li Chen, Jeffrey Gosian, Lara Skarf, Julie Paik, Amy Kind

**Affiliations:** 1 VA Boston Medical Center, Newton, Massachusetts, United States; 2 West Roxbury VA Hospital, West Roxbury, Massachusetts, United States; 3 VA Boston Healthcare System, Boston, Massachusetts, United States; 4 VA Boston Healthcare System, West Roxbury, Massachusetts, United States; 5 VA Hospital Madison, Madison, Wisconsin, United States

## Abstract

We developed the Supportive Coordinated Transitions of Care (SC-TraC) pathway at VA Boston to improve the quality of end-of-life (EOL) care. A nurse case manager (NCM) with training and experience in geriatrics and palliative care enrolled hospitalized patients with advanced illness (life expectancy < 2 years) who were not enrolled in hospice, and provided phone-based care coordination after discharge for up to 1 year. Our prior work found that SC-TraC patients were more likely to receive goal-concordant care, 60% more likely to enroll in hospice, twice as likely to die at home with hospice, and half as likely to die in an ICU, with no difference in survival. We worked with VA Geriatrics and Extended Care Data Analytics Center to calculate VA and Medicare/Medicaid cost data for a cohort of 104 SC-TraC cases and 104 carefully matched controls enrolled January 2017-June 2018, with follow-up through December 2019. Total cost data (VA + non-VA) was available for all patients up to 6 months following initial discharge. Difference in total cost per-patient was higher in SC-TraC patients at 30 days post-discharge (+ $3,258), but lower at 90 days (-$1,686) and 6 months (-$1,267). SC-TraC cost was substantially less in the last 30 days of life (-$-4,057). Cost differences were due to more home-based and less inpatient/institutional care in the SC-TraC cohort. This data suggests that the SC-TraC program promotes high value EOL care and is a financially sustainable model.

